# RNA Interference-Mediated Aurora Kinase A Gene Silencing Inhibits Human Glioma Cells Proliferation and Tumor Growth in Mice

**DOI:** 10.7150/jca.55791

**Published:** 2021-03-19

**Authors:** Ge Zhang, Guanghui Ren, Xin Zhao, Haibo Wang

**Affiliations:** 1 Department of Neurology, Luoyang Dong Fang Hospital, The Third Affiliated Hospital of Henan University of Science & Technology, Luoyang, Henan 471003, PR China.; 2 Institute of Cardiovascular Disease, Ruijin Hospital, Shanghai Jiao Tong University School of Medicine, Shanghai 200025, PR China.

**Keywords:** RNA interference, Aurora Kinase A, Glioma, Proliferation, Apoptosis, Tumor growth.

## Abstract

**Objective:** This study aims to explore the roles of Aurora Kinase A (Aurora A) in human glioma progression and relevant molecular mechanisms involved.

**Methods:** RNA interference (RNAi) technology was performed to silence the Aurora A gene in human glioma cell line U251 and U87. Western blot and real-time PCR were used to determine the protein and mRNA expression levels of Aurora A. Flow cytometry was performed to analyze the cell cycle distribution and MTT was used to examine the cell viability. Annexin V/FITC double staining and Hoechst 33258 staining were carried out to examine cell apoptosis. Xenograft tumor model was established to examine the effect of Aurora A siRNA on tumor growth *in vivo*.

**Results:** RNAi-mediated Aurora A gene silencing with specific short interfering RNA (siRNA) significantly decreased Aurora A protein and mRNA expression levels in human glioma cell line U251 and U87. Aurora A knockdown in glioma cells with siRNA strongly inhibited cell proliferation, along with the accumulation of cells in the G1, G2/M phase and decrease in S phase. Furthermore, the enhancement of cell apoptosis *in vitro* and the suppression of xenograft tumor growth *in vivo* were also observed after Aurora A silencing in U251 cell. In addition, Aurora A knockdown resulted in decreased expression of anti-apoptotic protein Bcl-2 and cell cycle protein Cyclin D1, while increased expression of pro-apoptotic factor caspase-3.

**Conclusion:** Aurora A can be used as a candidate targeting gene and inhibition of Aurora A is a potentially promising therapy for glioblastoma.

## Introduction

Glioblastoma (GBM) is the most aggressive and widespread brain tumor in adults. Patients that are diagnosed with GBM exhibit a median survival of merely 12 months, even with aggressive therapies such as chemotherapy, radiation and operative resection 1, 2. Furthermore, these high-grade gliomas relapse after operative resection with standard chemo- and radiation treatments. Several immunotherapies were applied for glioma cancer's therapy, including administrating antiglioma, growth factor receptor-specific antibodies and immune adjuvants, cytokine transduced glioma cell vaccines and *in vitro* stimulated tumor that infiltrated lymphocytes (TIL) and lymphokine-activated killer cells (LAK) 3. A few patients, however, were cured by these immunotherapeutic modalities occasionally. Despite recent progresses in both treatment strategies and diagnostic patterns, glioma continues to be one of the deadliest human cancers 4.

Three members are contained by serine/threonine kinases' Aurora family in mammalian cells, designated Auroras A, B and C, which have in common sequence homology's highest degree in their catalytic domains 5. Their cellular localization and expression pattern, however, are different markedly. Aurora A kinase is primarily expressed in the cell cycle's G2/M phase and regulates mitotic cell division in normal cells 6, 7, which is required for cell division and centrosome maturation 8. Aurora A is expressed in numerous different human normal tissues, which is, however, aberrantly overexpressed and has been involved in tumorigenesis recently, and correlated with poor prognosis in varieties of cancer patients 9, 10. Aurora Kinase A, a centrosome-associated serine/threonine protein kinase, is amplified and overexpressed in several types of human tumors, including glioblastoma. Previous study has demonstrated that Aurora A gene involved in mitotic processes, was associated with poor prognosis in glioblastoma. However, the roles of Aurora A in human glioma progression and relevant molecular mechanisms mediating these effects remain largely unknown.

Aurora A overexpression has been demonstrated in various of human cancers at multiple kinds of pathological stages, including hepatocellular carcinoma (HCC) 11, esophageal squamous cell carcinoma (ESCC) 12, gastrointestinal adenocarcinomas 13, ovarian cancer 9, head and neck squamous cell carcinoma (HNSCC) 10, and glioma 14, 15, etc., which has been associated with a poor prognosis of cancer patients. Furthermore, Aurora-B has also been found overexpressed in human gliomas 16. Aurora kinases' inhibition, especially Aurora A, therefore, is very urgent for treatment's implementation for human glioma. Several small molecule inhibitors have been recently developed revealing anti-proliferative effects on tumor cells in preclinical models. For example, it has been demonstrated that MLN8054 is a selective small-molecule Aurora A kinase inhibitor that has entered Phase I clinical trials for advanced solid tumors 17. Besides, some highly effective Aurora and pan-Aurora kinase inhibitors (AKI), which were capable to inhibit all aurora kinases, including PHA-739358, has entered phase 2 clinical trials for patients with advanced stages of prostate cancer and hematological malignancies 18, and SNS-314 19, VX-680 20 and AZD1152 21 show efficient anti-tumor activity. Therefore, the small molecules with an Aurora A inhibitory function may make it possible to reduce or inhibit the oncogenic activity of Aurora A 22. Previous studies have demonstrated that silencing of the gene that encodes Aurora A in tumor cells causes their growth inhibition and enhances the cytotoxicity of anticancer agents 23. These data powerfully suggest that Aurora A is one of the potential targets and fundamental cancer-related genes for cancer therapy 22, 23.

Aurora A gene expression was the only predictive factor. However, the biological functions of Aurora A in human glioma cells have yet to be well understood. In this study, we examined the effect of Aurora A silencing by RNAi on human glioma cell growth and apoptosis and relevant molecular mechanism involved.

## Materials and methods

### Cell culture

Human glioma cell line U251 and U87 were purchased from American Type Culture Collection (Manassas, VA, USA) and cultured in DMEM (Gibico, Grand Island, NY, USA) supplemented with 10% FBS (Gibico, Grand Island, NY, USA), penicillin (100 U/ml) and streptomycin (0.1 mg/ml). Cells were incubated at 37 ºC with humidified air containing 5% CO_2_.

### Short interference RNA transfection

The siRNA oligonucleotide sequence for Aurora A (NCBI: NM_003600) used was supplied by Dharmacon Research Inc. The sequence of siRNA: 5'-GCCGGUUCAGAAUCAGAAG-3'. The sequence of control siRNA: 5'-UUCUCCGAACGUGUCACGU-3'. siRNA transfection was performed with Lipofectamine 2000 (Invitrogen, NY, USA) according to the manufacturer's instructions. In brief, 24 h before transfection, U251 and U87 cells were trypsinized and transplated on a 6-well plate at 1*10^5^ cells/well in 2 ml of culture medium supplemented with DMEM and 10% serum without antibiotics for 24 h. Thereafter, 20 nM Aurora A siRNA or control siRNA diluted in 50 µl of Opti-MEM and 5 µl Lipofectamine 2000 diluted in 50 µl of Opti-MEM were preincubated for 5 min. The two mixtures were incubated for 25 min at room temperature for complex formation. 150 µl of Opti-MEM was added, then the entire mixture was added to each well. The cells were harvested 48 h after transfection.

### Real-time quantitative PCR

Total RNA was initially extracted from cultured glioma cells using TRIzol (Invitrogen) and treated with RNase-free DNase I. Standard reverse transcription reaction was carried out using a Promega M-MLV cDNA synthesis kit following the manufacturer's instructions. The real-time reverse transcription polymerase chain reaction was performed using the SYBR Green One-Step qRT-PCR kit (Invitrogen) according to the kit's procedure manual. GAPDH was used as an internal control. The sequences of the primers were as follows: GAPDH forward, 5'-TGACTTCAACAGCGACACCCA-3'; GAPDH reverse, 5'-CACCCTGTTGCTGTAGCCAAA-3'; Aurora A forward, 5'-AATGCCCTGTCTTACTGTCATTC-3'; Aurora A reverse, 5'-TCCAGAGATCCACCTTCTCATC-3'. The relative gene expression levels were calculated using the 2^-∆∆CT^ algorithm. Each assay was performed in triplicate and the experiment was repeated at least three times.

### Western blot

Glioma cells in the dishes were lysed directly with lysis buffer (50 mM Tris-HCl, 150 mM NaCl, 0.02% NaN3, 1% Triton X-100, 1 mM PMSF and 1 µg/ml aprotinin, 1 µg/ml leupeptin). The protein concentration was assayed by BCA Protein Assay (Pierce, Rockford, IL). Cell lysates containing equal amounts of protein were separated on 10% sodium dodecyl sulfate-polyacrylamide gel electrophoresis (SDS-PAGE) and electrotransferred onto nitrocellulose (NC) membranes (Millipore Billerica, MA). After overnight blocking with 5% non-fat dry milk in Tris-buffered saline (TBS) at 4°C, then the membranes were incubated with the following primary antibodies: anti-Aurora A, anti-Aurora B, anti-Aurora C (Santa Cruz, CA, USA), anti-Bcl-2, anti-Cyclin D1 and anti-Caspase-3 were purchased from Cell Signaling (Cell Signaling, Danvers, MA) and anti-β-actin (Sigma) at 4˚C overnight and horseradish peroxidase HRP-conjugated secondary antibodies (Jackson ImmunoResearch, PA) for 2 h at room temperature. The protein was visualized with ECL detection reagents (BioVision, CA, USA) and then was examined through scanning densitometry using Tanon Image System. Anti-β-actin antibody was used loading control. The experiment was repeated at least three times.

### MTT assay

Cell proliferation was examined using MTT as previously described 24. Briefly, U251 and U87 cells were seeded in quadruplicates on 96-well plates at a density of 1×10^5^ cells per well in 1 ml medium, then incubated in a 37°C humidified incubator for attachment. Cells were transfected with or without Aurora A siRNA /control siRNA after 24, 48 and 72 h at 37 °C in a 5% CO_2_ and 95% air-humidified environment, then 20 μl of MTT (5 mg/ml, Sigma, USA) was added to each well, and cells were incubated for another 4 h, then 250 μl of dimethyl sulfoxide (DMSO, Sigma, USA) was added and incubated for 30 min at room temperature for color development. The absorbance values were determined at 1, 2, 3 day in each group respectively. The optical density was determined on a microplate reader at 492 nm filter (Tecan, Groedig, Austria). Each assay was performed in triplicate and the experiment was repeated at least three times. The relative percentage of cell survival was determined as follows: mean absorbance from Aurora A siRNA or control siRNA group/mean absorbance from parental group × 100%.

### Cell cycle analysis

At 3 days after transfection, U251 and U87 cells from parental, Aurora A siRNA or control siRNA treatment group, respectively were collected by centrifugation, washed thrice with cold PBS, and fixed with 70% ethanol at 4°C overnight. The fixed cells were resuspended in PBS containing 10μg/mL propidium iodide (PI, Sigma, USA) and 100μg/mL RNase A, then incubated at 4℃ for at least 30 min avoiding light to eliminate the endogenous RNA. Cell cycle distribution was determined using flow cytometry (Becton-Dickinson FACScan). Each assay was performed in triplicate and the experiment was repeated at least three times.

### Apoptosis assay

Apoptosis was detected with Annexin V-FITC kit (BD Bioscience, NY, USA) according to the manufacturer's instructions. In brief, at 3 day after transfection, U251 cells from parental, Aurora A siRNA or control siRNA group were trypsinized, collected and washed with cold PBS and centrifuged. Cell pellets were resuspended in cold binding buffer. Annexin V-FITC (1.25 μl /0.5 ml) and propidium iodide (PI) solution (10 μl/0.5 ml) were then added. The tubes were incubated for 15 min in the dark on ice before analysis by flow cytometry (Becton-Dickinson FACScan). Apoptosis was also determined through the fragmented nuclear staining. Cells from various treatment groups were washed with PBS and fixed with ice-cold methanol for 30 s. Cells were stained with Hoechst 33258 (Invitrogen, Grand Island, NY, USA) for 30 min in the dark before morphological analysis under the fluorescence microscope (Olympus). Each assay was performed in triplicate and the experiment was repeated at least three times.

### Liposomal siRNA preparation

siRNA for *in vivo* delivery was incorporated into a neutral liposome (1,2-dioleoyl-sn-glycero-3-phosphatidylcholine; DOPC). siRNA and DOPC were mixed in the presence of excess t-butanol at a ratio of 1:10 (w/w) as described previously 25, 26. Tween 20 was added to the mixture, which was then vortexed, frozen in an acetone/dry ice bath, and lyophilized. Before *in vivo* administration, this preparation was hydrated with normal (0.9%) saline at a concentration of 15 µg/mL to achieve the desired dose in 150 to 200 µL per injection.

### *In vivo* xenograft tumor model

The female nude mice (4 - 6 weeks old, Animal Center of the Chinese Science Academy) were housed in specific pathogen-free conditions. 5x10^6^ U251 cells in 0.1 ml of PBS solution were injected subcutaneously into the right flank of each nude mice. After 7 days, when the tumor volume was about 100 mm^3^, the mice were randomly subjected to three groups (n = 6) for daily intra-tumor injection of liposomal/Aurora A siRNA (5 μg/100 μl), liposomal/control siRNA (5 μg/100 μl) and liposomal/PBS (100 μl as blank) for 21 days. The perpendicular diameters were measured with a caliper every 3 to 4 days, and tumor volume was calculated according to the formula: (V) = a x b^2^ x 0.5. After 3 weeks, the mice were sacrificed, and their tumor tissues were harvested. All animal experiments were approved by the Ethics Committee for Animal Use of our hospital.

### Statistical analysis

All results were presented as mean ± standard deviation (SD). Differences among experimental groups were analyzed for statistical significance by one-way analysis of variance (ANOVA) followed by Bonferroni post hoc tests and the nonparametric tests (Tukey test), Value of *P* < 0.05 was considered as significant.

## Results

### Specific knockdown of Aurora A in glioma cell line

**Figure 1:**
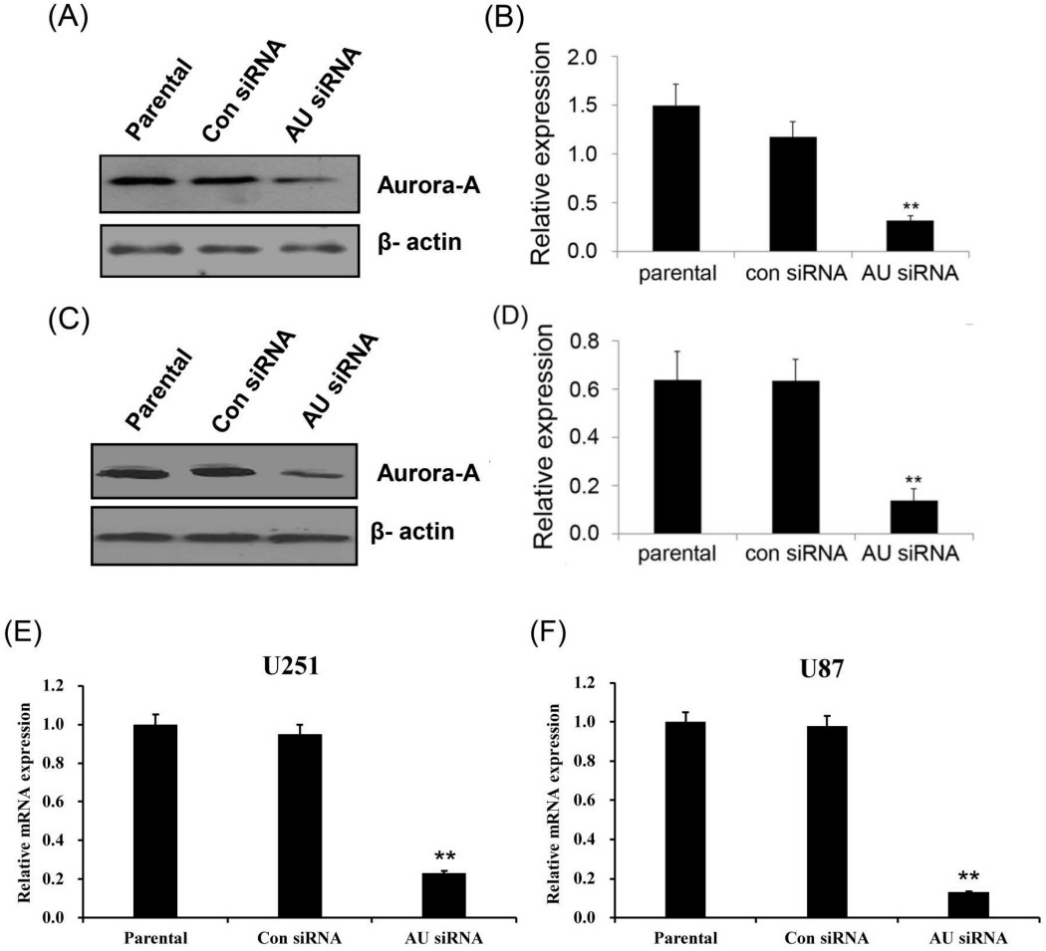
Aurora A silencing with siRNA inhibited its expression in glioma cells. Aurora A protein expression was examined by Western Blot in U251 cells (A) and U87 cells (C). Aurora A protein quantification in U251 cells (B) and U87 cells (D) were also presented, respectively by densitometric analysis of band area. β-actin served as an internal control. Aurora A mRNA expression was examined by real-time qPCR in U251 cells (E) and U87 cells (F). The expression levels of mRNAs were normalized to that of GAPDH mRNA. The experiments were performed three times with consistent and repeatable results. Each bar represents the mean ± SD, and ** p < 0.01, AU indicates Aurora A, con siRNA indicates control siRNA.

Western blot and real-time qPCR analyses were performed after 48 hours transfection. RNAi-mediated gene silencing significantly reduced the protein and mRNA expression of Aurora A by ~ 80% and ~ 75% as compared to the control siRNA treatment in the U251 **(Fig. 1A, B, E)** and U87 **(Fig. 1C, D, F)** cell lines, respectively. In view of the homology of the three Aurora kinases, Aurora A, Aurora B and Aurora C, we documented the specificity of the Aurora A silencing, the results showing that the expression of Aurora B and Aurora C are not affected by Aurora A siRNA **(Supplemental Fig. 1)**. These results indicated that Aurora A expression was significantly inhibited by siRNA, neither Aurora B nor Aurora C.

### Effects of Aurora A knockdown on glioma cells proliferation and cell cycle distribution

**Figure 2:**
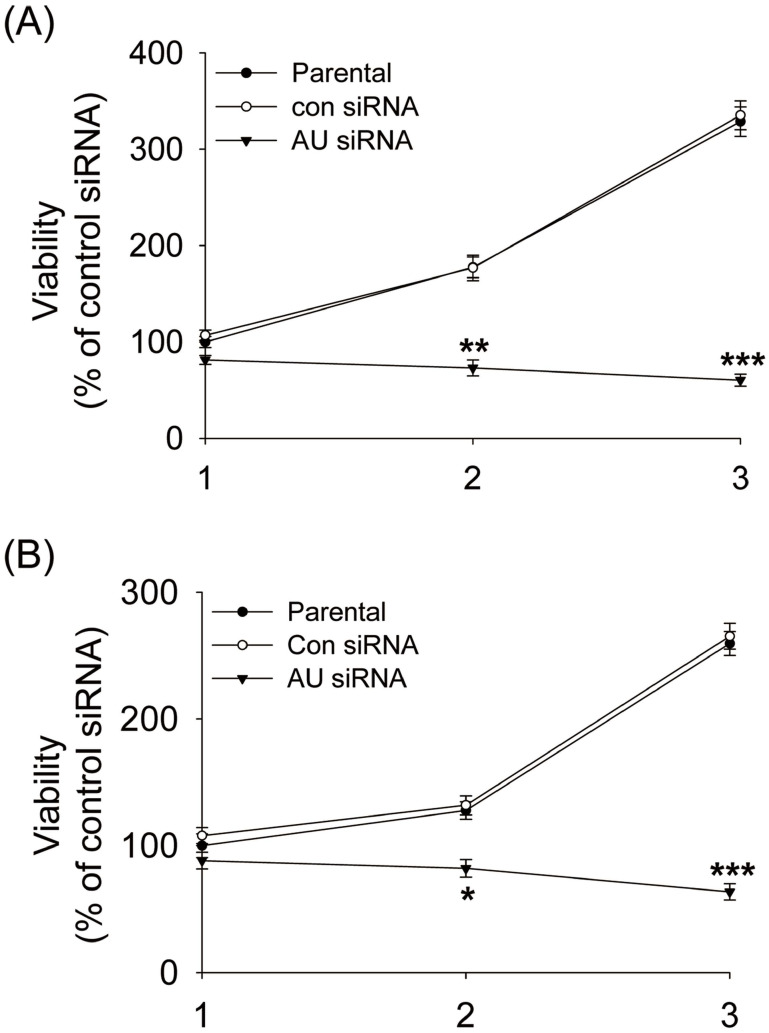
Aurora A silencing with siRNA inhibited cell proliferation. U251 (A) and U87 cells (B) were transiently transfected with Aurora A siRNA, control siRNA or no-treated (parental) for 24, 48, and 72 hours respectively, MTT assay was performed to assess cell proliferation. The experiment data was expressed as mean ± SD from triplicate and repeated three times independently. * p < 0.05, ** p < 0.01, *** p < 0.001, AU indicates Aurora A, con siRNA indicates control siRNA.

To further investigate the roles of Aurora A in glioma cells, we determined the effects of Aurora A silencing on glioma cells proliferation and cell cycle distribution. Our data showed that Aurora A silencing significantly suppressed cell proliferation in comparison with the control siRNA or non-treatment (parental) group both in U251 **(Fig. 2A)** and U87 cells **(Fig. 2B)**.

**Figure 3:**
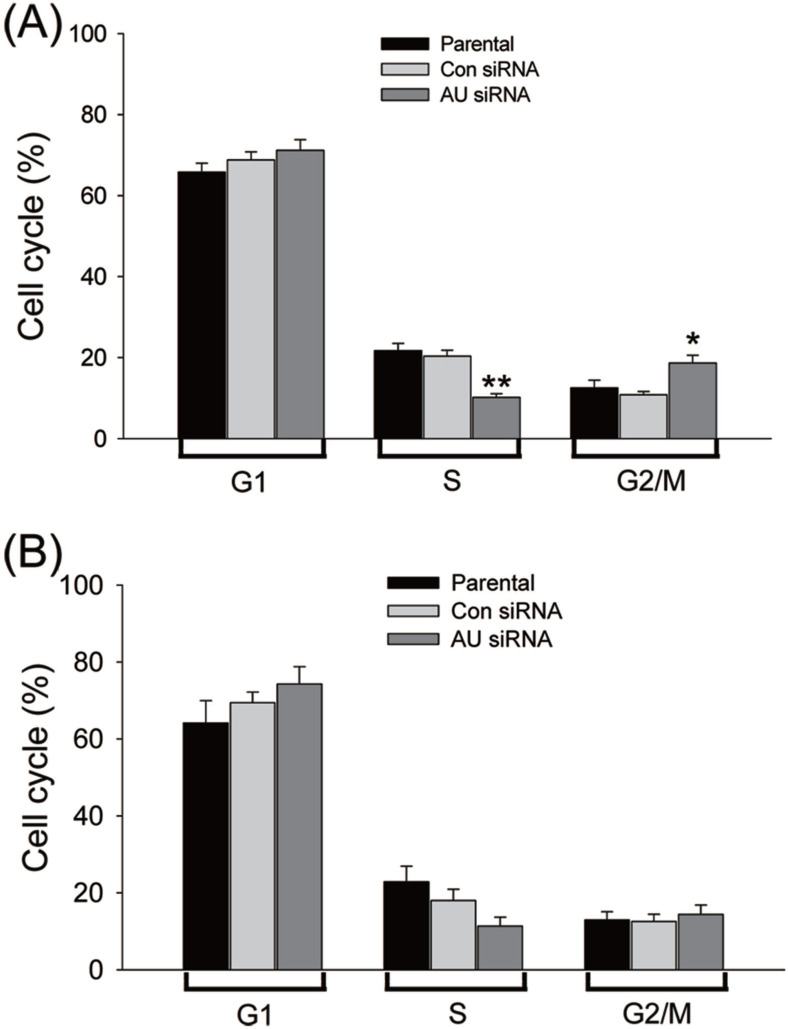
Effects of Aurora A silencing with siRNA disrupted cell cycle progression. Glioma U251 (A) and U87 cells (B) were collected 72 hours after Aurora A siRNA, control siRNA transfection or non-treatment (parental), respectively and subsequently assayed for their DNA content by flow cytometry. The experiments were repeated at least three times independently and the data were expressed as mean ± SD from triplicate. * p < 0.05, ** p < 0.01, AU indicates Aurora A, con siRNA indicates control siRNA.

Aurora A plays an important regulatory role for the bipolar spindle formation. Thus, it is crucial for precise chromosome segregation 27, 28. We supposed that the proliferation inhibition of the glioma cells by RNAi-mediated Aurora A gene silencing was caused by disruption of cell cycle transition. To explore this possibility, the DNA content of cell populations reflecting the cell cycle distribution after Aurora A knockdown was assayed. A significant reduction in the S phase cells after Aurora A knockdown than that of the other two groups was found in the U251 **(Fig. 3A)** but not in the U87 cells **(Fig. 3B)**. This also applies to the percentage of G2/M cells which were significantly varied in the U251, but not in U87 cells. The data indicated that the RNAi-mediated silencing of Aurora A not only inhibited glioma cells proliferation but also affected cell cycle distribution.

### Effect of Aurora A knockdown on glioma cells apoptosis

**Figure 4:**
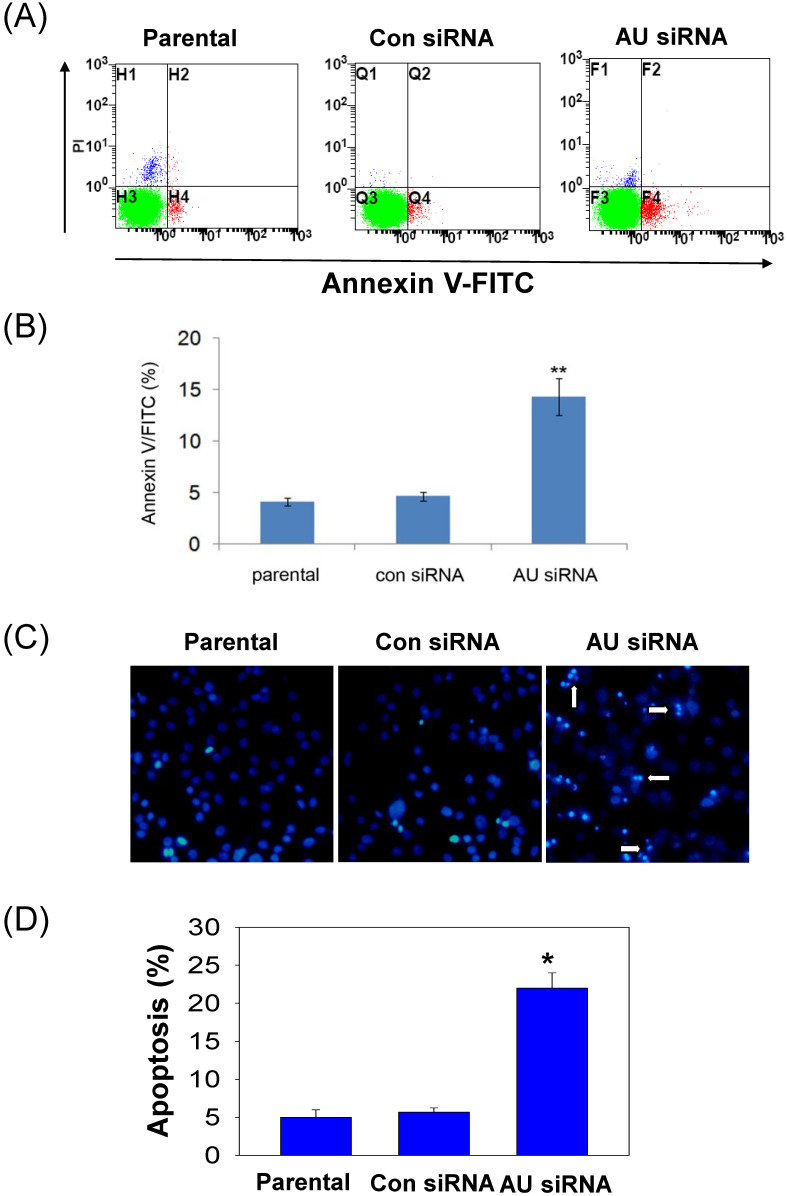
Effect of Aurora A silencing with siRNA on U251 cell apoptosis. U251 cells were transiently transfected with Aurora A siRNA, control siRNA or no-treated (parental). After 72 h, cell apoptosis was assayed by Annexin V/FITC double staining and by nuclear morphology changes examination with Hoechst 33258 staining, as described in the materials and methods section. (A) The representative images of U251 cell apoptosis from different treatment groups were shown. (B) The apoptosis was summarized as the percentage of apoptotic cell numbers to total cell numbers. (C) Apoptotic cells showed typical nuclear fragmentation (white arrows) and the quantitative data showed the apoptotic ratio in various treatment groups (D). The experiments were repeated three times independently and the data were expressed as mean ± SD from triplicate. * p < 0.05, ** p < 0.01, AU indicates Aurora A, con siRNA indicates control siRNA.

To further confirm our findings, Annexin V/FITC and Hoechst 33258 staining assay were performed to examine the effect of RNAi-mediated Aurora A silencing on glioma cells apoptosis. Apoptosis was determined using an Annexin V/FITC kit according to the manufacturer's protocol. Aurora A siRNA significantly increased U251 cell apoptosis, neither control siRNA nor untreated group (parental, **Fig. 4A**). The rate of cell apoptosis in Aurora A siRNA accounted for ~ 15%, almost 3-fold than that of the other two groups **(Fig. 4B)**, indicating the remarkably increased cell apoptosis by RNAi.

To clarify the changes of apoptotic cell morphology after Aurora A knockdown, Hoechst 33258 staining was performed to observe nuclear changes and apoptotic body formation. Similar result that much more apoptotic body was observed after Aurora A siRNA treatment than that of the other two groups in U251 cells **(Fig. 4C)**. Also, the numbers of the apoptotic cells, as shown in **Fig. 4D**, quantitated the results of nuclear staining from different treatment groups.

**Figure 5:**
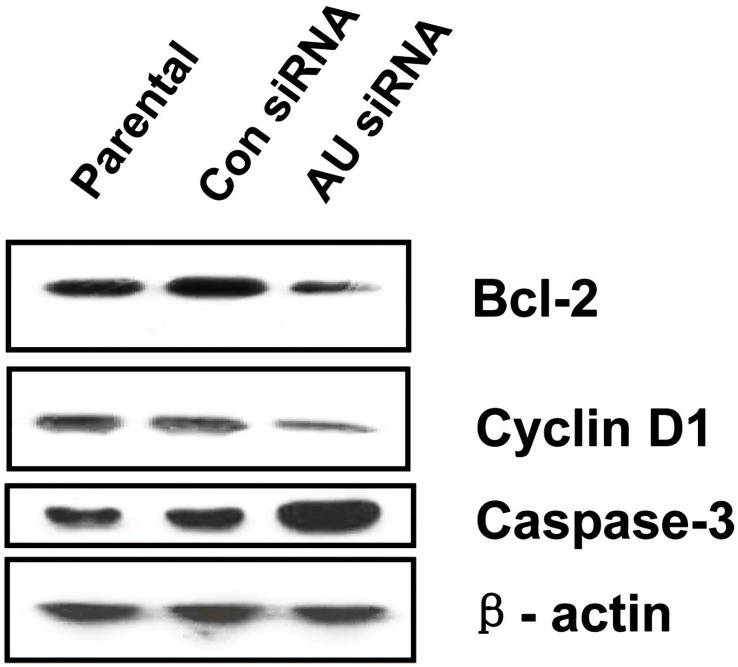
Effects of Aurora A silencing with siRNA on the expression of Bcl-2, Cyclin D1 and Caspase-3 in U251 cells. Total proteins from Parental, control siRNA and Aurora A siRNA treatment groups were extracted and analyzed by Western blot. β-actin was used as loading control. The experiments were repeated three times independently. Aurora A knockdown in U251 cells resulted in decreased expression of Bcl-2 and Cyclin D1, while increased expression of caspase-3 compared with the other two groups. AU indicates Aurora A; con siRNA indicates control siRNA.

To illustrate the proliferation- or apoptosis-related molecules involved in RNAi-mediated Aurora A gene silencing leading to cell proliferation inhibition and cell apoptosis promotion, we analyzed the expression levels of cell cycle-related gene Cyclin D1, anti-apoptotic factor Bcl-2 and pro- apoptotic factor caspase-3 with western blot. As shown in **Fig. 5**, the Cyclin D1 and Bcl-2 protein levels were decreased, but the caspase-3 expression level was obviously increased in Aurora A siRNA treatment group compared with the other two groups.

### Knockdown of Aurora A suppresses tumor growth *in vivo*

To explore the RNAi-mediated Aurora A silencing in glioma cells would affect the tumor growth *in vivo*, aliquots of 5×10^6^ U251 cells were inoculated into the immunodeficient female nude mice and the tumor growth was monitored. No signs of possible toxicity or death were observed during the study period of time.

**Figure 6:**
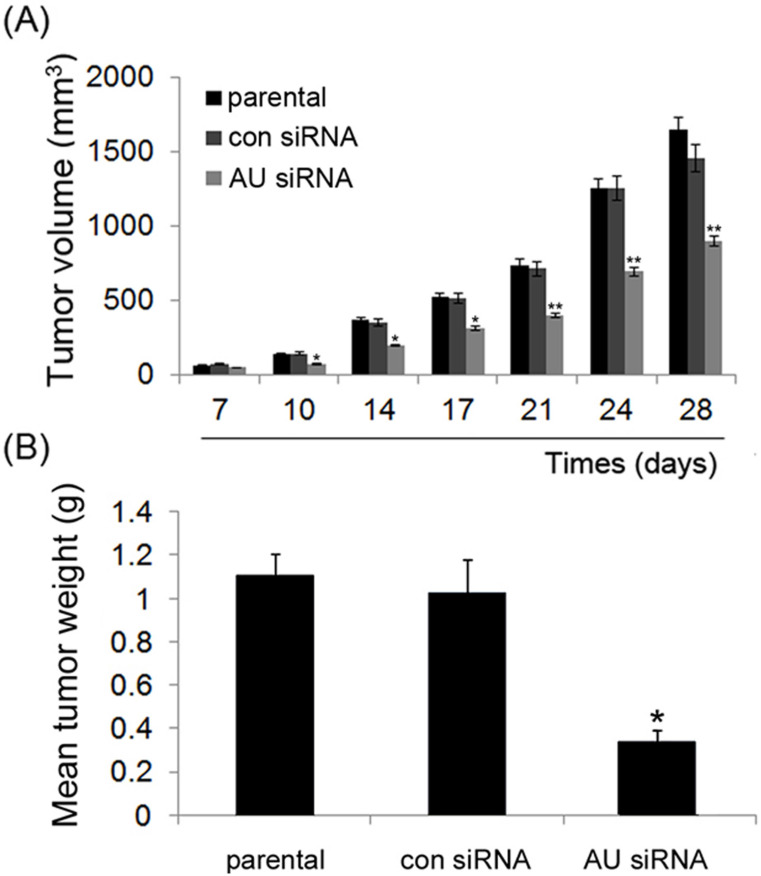
Effect of Aurora A silencing with siRNA on xenograft tumor growth *in vivo*. Intra-tumor injection of PBS (parental), Aurora A siRNA/DOPC (AU siRNA), or control siRNA/DOPC (con siRNA), respectively was performed. siRNA treatment caused a significant decrease in tumor volume (A) and tumor weight (B) as compared to control siRNA or PBS treatment group. Each bar represents the mean ± SD. n = 6, and * p < 0.05, ** p < 0.01, AU indicates Aurora A, con siRNA indicates control siRNA. DOPC, purchased from Roche, was used for siRNA *in vivo* delivery. In brief, DOPC and siRNA oligos were mixed in the presence of excess tertiary butanol at a ratio of 1:10 (w/w), Tween 20 was added to the mixture in a ratio of 1:19 (Tween 20:siRNA/DOPC), then the siRNA oligos were incorporated into DOPC, as described in previous study 25, 26.

As illustrated in **Fig. 6A**, during the course of treatment, all xenograft tumor volumes increased, but significant lower growth rates appeared in the tumors that were treated with Aurora A siRNA compared to those treated with control siRNA or non-treated (parental) group at the end of the experiment. Also, at the end of the experiment, the mean tumor weight also decreased treated with Aurora A siRNA as compared to those of two other groups **(Fig. 6B).** Our data indicated that RNAi-mediated Aurora A gene silencing played a strong anti-tumor effect *in vivo*, consistent with the results of the cell proliferation *in vitro*, as shown in Fig. 2A, B.

## Discussion

Aurora Kinases plays an important role in maintaining appropriate chromosome segregation and have been related with various of cancers. Particularly, high expression level of Aurora A has been abnormally found in a large number of cancers including breast, ovarian, and liver cancer as well as glioma when compared to their respective normal tissues 29, 30. Previous studies have shown that Aurora A might act as a potential target for anticancer treatment in various types of cancers 17, 23, 30, 31. Whereas, the role of Aurora A in glioma cell growth has not yet been understood. Aurora kinase inhibitors have been developed for more than one decade. The recent research data indicated that aurora kinase inhibitors promote apoptosis via mitochondrial pathway 20, 32, 33. However, specificity, safety, and efficacy currently remain the most important challenges in the use of Aurora A inhibitors for cancer treatment 34, 35. Furthermore, the ATP-competitive property of the Aurora A small molecule inhibitors also reduces their specificity due to their off-target effects on other kinases. Meanwhile, more and more studies have shown that specific inhibition of Aurora A by RNA interference can effectively inhibit tumor growth 36-38. Therefore, the development of selective siRNA is a prospective strategy for targeting Aurora A mRNA specifically expressed in glioblastoma, and RNAi is becoming a routine application for cancer treatment *in vivo*. In the present study, we employed the RNAi technology to knockdown the expression of Aurora A and analyze its phenotype, to explore the possibility of Aurora A as a therapeutic target. We demonstrated that knockdown of Aurora A using siRNA treatment strategy in glioma cells suppressed cell growth *in vivo* and *in vitro*.

Moreover, we showed that the knockdown of Aurora A significantly enhanced glioma cells apoptosis. The previous publications showed that the inhibition of aurora kinase could induce cell apoptosis, which is dependent on the polyploidization of cells 39, 40. In our work, we found that inhibition of Aurora A induced the strong accumulation in G2/M phase. Our findings are in good agreement with the previous studies by Hata et al. 23 that RNAi targeting Aurora kinase suppressed pancreatic cancer cells growth and induced cells to accumulate in G2/M phase. In addition, El-Sheikh et al. 29 suggested that RNAi-mediated knockdown of Aurora kinase A can arrest the cell cycle and reduce the viability of medulloblastoma cells. All of these observations, together with our results indicate that Aurora A is an attractive candidate target for cancer treatment.

It is worth noting that, in accordance with the results obtained *in vitro*, inhibition of Aurora A obviously decreased tumor growth *in vivo* than that in the control groups (Fig. 6). This result suggests that the silence of Aurora A gene could significantly inhibit the tumor cell growth *in vivo*, and also suggests that the knockdown strategy will be applicable in prohibiting the cancer's progression *in vivo*.

Studying molecular alterations is important in cancer research 41, 42. As is well known, the oncoprotein Bcl-2 functions to inhibit or delay the induction of apoptosis in numbers of systems. Cyclin D1, one of cell cycle regulatory proteins, plays a key role in determining cell cycle transition from G1 to S phase. Whereas, caspase exists in the form of zymogens in healthy cells, which can be activated by interacting with specific adaptor proteins to promote conformational changes and autocatalytic processes, or proteolysis by already active caspase 43-47. In our present study, we observed that the expression of Bcl-2 and Cyclin D1 were reduced by RNAi targeting against Aurora A in the U251 cells, on the contrary, the expression level of caspase-3 was upregulated obviously compared with the control groups (Fig. 5). As we all know, Bcl-2 and its homologues cause cell survival by blocking the activation of caspase 48. Therefore, our results suggest that the cell proliferation inhibition by Aurora A knockdown was produced by modulating cell anti-apoptosis associated genes. This is also in accordance with the recent study indicating that Aurora A down-regulation resulted in increased caspase activity and enhanced cell apoptosis 30, 41. Our work supported the claim that Aurora A targeted therapy provides an opportunity for treatment of glioma in the future, especially in high-risk patients. However, the molecular mechanism of Aurora A siRNA inducing apoptosis remains to be further elucidated.

Our study demonstrated that knockdown of Aurora A expression by siRNA strongly suppressed glioma cells growth *in vivo* and cell proliferation *in vitro*, as well as induced glioma cells apoptosis, indicating that Aurora A could be considered as a candidate target in gene therapy. However, the precise regulation mechanisms of Aurora A in glioblastoma remain poorly clear. Therefore, we will use new high-throughput “Omics” technologies such as genomics and epigenomics to study effect of Aurora A in glioblastoma with further analyses. In the future, we intend to analyze the interaction between transcription factors and chromosomes by HiC and ATAC-seq methods and find Aurora A and transcription factors related to key regions of chromosomes, and then further confirm the regulatory relationship between transcription factors-Aurora A-target genes through co-IP technology. Consequently, establishment of these regulatory relationships may open new perspectives to optimize therapies targeting epigenetic mechanisms of Aurora A in glioblastoma. With the continuous deepening of Aurora A kinase family research, the effects of Aurora A on mitosis and human malignancies will further clear, making it becomes a most promising tumor therapeutic targets.

## Conclusions

Aurora A can be used as a candidate targeting gene and inhibition of Aurora A is a potentially promising therapy for glioblastoma.

## Supplementary Material

Supplementary figures and tables.
