# Supplementary material for: RNA Interference-Mediated Aurora Kinase A Gene Silencing Inhibits Human Glioma Cells Proliferation and Tumor Growth in Mice
Source: J Cancer. 2021 Mar 19;12(10):3024–32. doi: 10.7150/jca.55791 (PMC8040878; doi:10.7150/jca.55791)
Supplement: Supplementary file 1 — Supplementary figures and tables. [file jcav12p3024s1.pdf]

Supplementary materials

Article title

RNA Interference-Mediated Aurora Kinase A Gene Silencing Inhibits Human Glioma Cells Proliferation And Tumor Growth In Mice

Running title: Aurora A silencing inhibits glioma growth

Author names and affiliations

Ge Zhang[1], Guanghui Ren[1], Xin Zhao[1], Haibo Wang[2, #]

[1] Department of Neurology, Luoyang Dong Fang Hospital, The Third Affiliated Hospital of Henan University of Science & Technology, Luoyang, Henan 471003, PR China

[2] Institute of Cardiovascular Disease, Ruijin Hospital, Shanghai Jiao Tong University School of Medicine, Shanghai 200025, PR China

[#] **Correspondence to:** Haibo Wang

Street address: No. 197 Ruijin 2nd Road, Huangpu District, Shanghai, China. Postal code: 200025

Email Address: wanghaibo@sjtu.edu.cn

Phone: 86-021-64370045

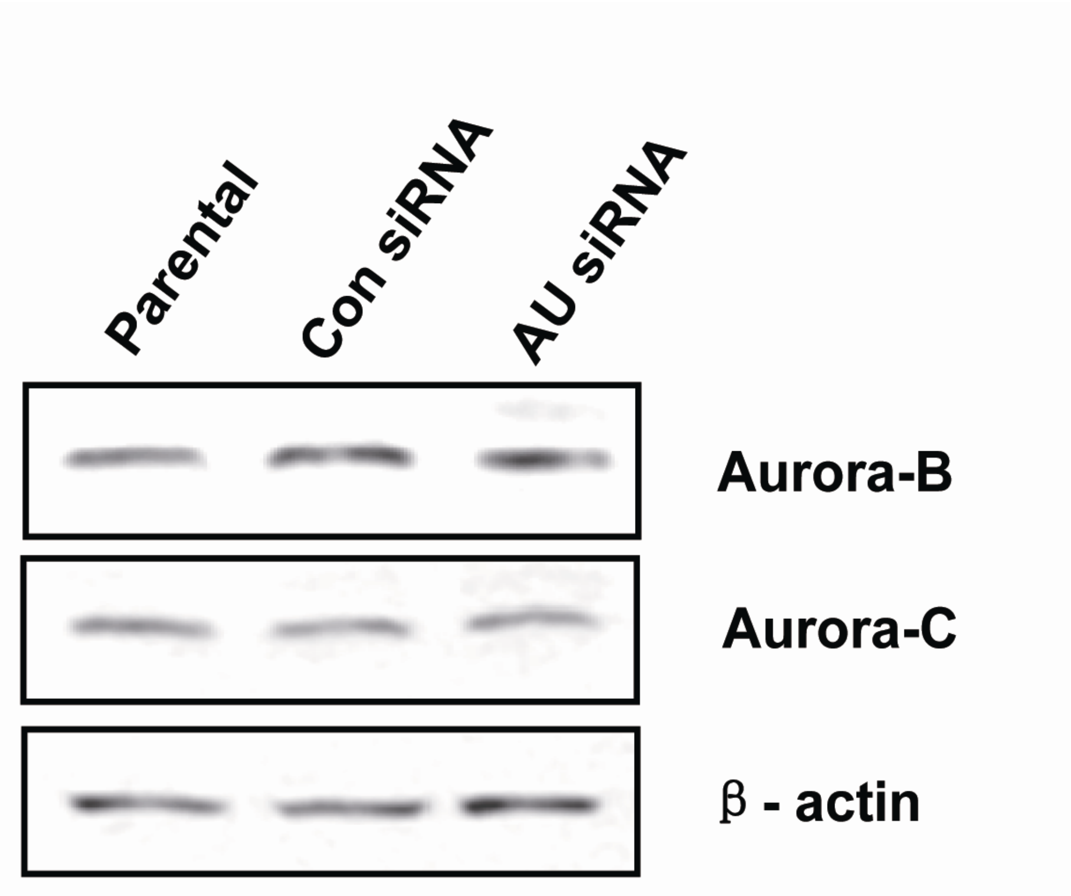

Supplemental Fig. 1 Effects of Aurora A silencing with siRNA on Aurora B and Aurora C kinases expression in glioma cells. U251 cells were transiently transfected with Aurora A siRNA, control siRNA or no-treated (parental) for 48 h. Aurora B and Aurora C protein expression were examined by Western Blot with an Aurora B or Aurora C specific antibody, respectively. β-actin served as an internal control.
